# The nocebo effect challenges the non-medical infliximab switch in practice

**DOI:** 10.1007/s00228-018-2418-4

**Published:** 2018-01-24

**Authors:** N. W. Boone, L. Liu, M. J. Romberg-Camps, L. Duijsens, C. Houwen, P. H. M. van der Kuy, R. Janknegt, R. Peeters, R. B. M. Landewé, B. Winkens, A. A. van Bodegraven

**Affiliations:** 1 Department of Clinical Pharmacy, Pharmacology and Toxicology, Zuyderland Medical Centre, PO Box 5500, NL, 6162 BG Heerlen, Sittard-Geleen The Netherlands; 2 Department of Gastroenterology, Geriatrics, Internal and Intensive Care Medicine (Co-MIK), Zuyderland Medical Centre, Heerlen, Sittard-Geleen The Netherlands; 3 Department of Clinical pharmacy, Erasmus Medical Centre, Rotterdam, The Netherlands; 4 Department of Rheumatology, Zuyderland Medical Centre, Heerlen, Sittard-Geleen The Netherlands; 5 Amsterdam Rheumatology & Immunology Centre, Amsterdam-Zuidoost, The Netherlands; 6 Department of Methodology and Statistics, Care and Public Health Research Institute (CAPHRI), Maastricht University, Maastricht, The Netherlands

**Keywords:** Inflammatory bowel disease, Crohn’s disease, Ulcerative colitis, Rheumatoid arthritis, Psoriatic arthritis, Ankylosing spondylitis, Non-medical switch, Nocebo-effect

## Abstract

**Background:**

In clinical practice, non-medical switching of biological medication may provoke nocebo effects due to unexplained deterioration of therapeutic benefits. Indication extrapolation, idiosyncratic reactions, and interchangeability remain challenged in clinical practice after biosimilar approval by the European Medicines Agency. The principle of “first do no harm” may be challenged in a patient when switching from originator to biosimilar biological.

**Aim:**

To describe the 1-year results of a pragmatic study on infliximab biosimilar implementation in immune-mediated inflammatory disease patients on the basis of shared decision-making under effectiveness and safety monitoring.

**Methods:**

Inflammatory bowel disease and rheumatology patients on infliximab originator were converted to infliximab biosimilar after providing informed consent. Nocebo response patients were monitored after switch back to originator. Linear mixed models were used to analyze continuous endpoints on effectiveness and laboratory outcomes to determine significance (*P* ≤ 0.05) of change over time after switching.

**Results:**

After inviting 146 patients, a group of 125 patients enrolled in the project over time, respectively, 73 Crohn’s disease, 28 ulcerative colitis, nine rheumatoid arthritis, ten psoriatic arthritis, and five ankylosing spondylitis patients. No statistically significant changes in effectiveness and safety were observed in any of the indications after a median of 4 infusions in 9 months of study. An overall nocebo response of 12.8% was found among the patients during a minimal observation period of 6 months after the transition to biosimilar infliximab. The overall nocebo response rate did not differ between the studied indications.

**Conclusions:**

In inflammatory bowel disease and rheumatological patients, similar effectiveness and safety were demonstrated on the transition into infliximab biosimilar. In our series, patient empowerment and registration of treatment outcomes delineated biosimilar transition, an approach that hypothetically could reduce nocebo response rates which are relevant to account for regarding biosimilar implementation.

**Electronic supplementary material:**

The online version of this article (10.1007/s00228-018-2418-4) contains supplementary material, which is available to authorized users.

## Introduction

In 2013, the European Medicines Agency approved monoclonal antibody infliximab biosimilar, based on pharmacological similarity and safety results. In the approval process of infliximab, biosimilar preclinical similarity testing was emphasized, followed by a limited program of clinical comparability confirmation studies in rheumatoid arthritis and ankylosing spondylitis patients [1]. Based on the results of these adapted registration studies, comparable effectiveness and safety were concluded for infliximab biosimilar [2]. Nevertheless, clinical concerns were being raised concerning indication extrapolation, safety and idiosyncratic adverse drug reactions, and issues of substitution and interchangeability.

More recently, it was shown in the NORSWITCH study that non-inferiority of infliximab biosimilar was likely if compared to infliximab originator against a margin of 15% [3].

Both this study as well as the European Medicines Agency approval remain however challenged as being sufficient for gaining full trust in the treatment practice of medical doctors and their patients, subsequently. Clinical concerns in case of applying (biosimilar) biologicals are partly based on the extrapolation of the indication denying potential variance in pharmacokinetics and pharmacodynamics over time [4]. Indeed, one may raise concerns with respect to non-medical switching and/or interchangeability whether the principle of “first do no harm (primum nil nocere)” is challenged in an individual patient on treatment with a(n) (originator) biological (with for that person proven and documented effectiveness and safety). Therefore, in Dutch treatment guidelines, it has been stated that biosimilars should only be implemented in naïve patients or in treated patients following informed consent under close monitoring of effectiveness and safety, preferably in a clinical study [5–7].

In clinical practice, non-medical switching may induce nocebo effects, i.e., unexplained detrimental therapeutic effects. This underlines that in any switching study so far, the second biological drug, thus even biosimilar ones, exerts less therapeutic benefit due to unexplained but nevertheless existing patients’ concerns about value and effect of a drug [8]. For this reason, shared decision-making between doctor and patient remains essential, specifically when pure economic aspects of biological use may tend to prevail. Relevant practical issues that cannot be fully tackled or facilitated in practice are the traceability of biologicals on the level of medication batch numbers, and the use of the correct brand name (usually being the generic name, e.g., infliximab, more than Remicade®, Remsima®, Inflectra®, or Flixabi®) which is necessary for proper pharmacovigilance in order to designate any adverse drug reactions to the correct drug brand or batch or sequence of various drug brands being applied in a single, unique patient.

These uncertainties, accompanied by medical concerns, have to be tackled by a critical healthcare professional who is not always familiar with the pharmacological finesses of large protein drugs or their biosimilars. Therefore, biopharmaceutical products should be implemented with appropriate criticism with concomitant monitoring of treatment quality, and patients’ benefit, allowing for evidence-based treatment decision, preferably in a setting with shared decision-making between physician and patient, more than on cost efficiency only.

In this paper, we aimed to describe infliximab biosimilar implementation in IBD and rheumatology practice, guided by effectiveness and safety monitoring, and particularly focusing on the nocebo response to quantify patients’ acceptance. We present the 1-year results of a pragmatic trial performed in the period between July 2016 and April 2017.

## Patients and methods

The switch protocol of originator to biosimilar infliximab was written by a consensus team (NB, MR, RP, AAvB) with mandate of the team of gastroenterologists, rheumatologists, and clinical pharmacists familiar with the concept of biosimilars. The protocol was reviewed by the local medical ethics committee as an observational study in clinical routine and was in accordance with guidelines for non-medical switching into biosimilars of the Dutch Society of Gastroenterologists, Dutch Association of Rheumatology, the Dutch Society for Consultants (FMS), and the Dutch Medicine Evaluation Board (CBG) [5–7]. Patients with Crohn’s disease (CD), ulcerative colitis (UC), rheumatoid arthritis, psoriatic arthritis, and ankylosing spondylitis were treated according to routine practice and were followed during 7 infusions in this observational study. In this report we present the 1-year results.

### Patient population

The therapy expectation following a possible non-medical switch was communicated with patients by written documentation first (provided as electronic supplement 1) and was accompanied by oral clarification by the patients’ treating physician or nurse practitioner when requested.

Patients providing informed consent voluntary agreed with transition into infliximab biosimilar, this formal procedure was used to emphasize that a non-medical driven change of treatment was introduced in a shared decision-making fashion. Participating patients were treated with infliximab according to their usual dose and routine treatment interval. If patients were switched back to originator infliximab for specific reasons, they were still monitored during the project’s follow-up period. The follow-up of patients was censored after switching to other (biological) therapy due to non-response on infliximab.

### Consultants

The gastrointestinal and rheumatology consultants from a large regional, referral hospital actively identified potential participants from electronic patient records and by recruitment on the day-care facility for infliximab administration. Positive identification for participation was based on indication, adulthood, and the continuous treatment with infliximab originator. Drug prescribing was managed via the electronic medication system by brand name and the laboratory measurements during the project were pre-ordered via the electronic patient dossier. Patients that were included visited consultants and nurses during the follow-up on alternating (routine) basis.

### Data collection

A dedicated project database using Microsoft Excel (2013 Edition; Microsoft Corp, Redmond, WA, USA) was designed for the retrospective collection of treatment outcome parameters. Data on effectiveness was acquired via an online electronic patient questionnaires platform (Sermos e-communication in healthcare) in which patients completed the form during the infusion. This portal meets the data requirements with ISO 9001/ISO 27001 certification. Safety data were kept in the electronic patient file by the gastroenterologists and rheumatologists and was related to the most recent date of infusion. Laboratory measurements were ordered according to routine care. IFX was prepared according to standard local protocol with registration of individual batch numbers with weekly update [9, 10]. Patients and treatment characteristics and safety data were derived from the electronic patient questionnaires platform, the patient’s file, medication prescription system, and the laboratory system—the database.

### Data acquisition

Monitoring parameters related to key issues in clinical biosimilar implementation were defined as follows and measured before and after infliximab transition. Effectiveness and safety were documented during every infusion visit. In both IBD indications, pragmatically, patient’s assessment of disease activity on a 0–10 scale ranging from worse to good was used. The Disease Activity Score 28-erythrocyte sedimentation rate (DAS28-ESR) was used for effectiveness measurements in rheumatoid arthritis and psoriatic arthritis [11]. The Bath Ankylosing Spondylitis Disease Activity Index (BASDAI) was used for outcome measurements in ankylosing spondylitis [12].

Generally, well-accepted laboratory assessments representative for disease activity in the investigated indications were chosen. Pragmatically, in both IBD indications, fecal calprotectin as a mucosal disease activity measure and C-reactive protein (CRP) were analyzed. In rheumatology indications, both CRP and the erythrocyte sedimentation rate (ESR) were assessed. Pharmacokinetics and immunogenicity were monitored by measuring serum infliximab trough concentrations and the formation of neutralizing antibodies against biosimilar or originator infliximab at trough. The reporting of these test results was with delay for logistical reasons. Infliximab serum concentration was measured using a validated enzyme-linked immunosorbent assay (Sanquin Laboratories, Amsterdam, NL) [13]. The free fraction of serum neutralizing antibodies to infliximab was analyzed using a validated radio immune-assay [14]. All laboratory measurements were assessed per protocol before the first, second, fourth, and seventh (biosimilar) infliximab infusion. In Table 1, an overview of the assessments per indication is provided.

**Table 1:** Demographics and baseline values of Crohn’s disease (CD), ulcerative colitis (UC), rheumatoid arthritis (RA), psoriatic arthritis (PsoA), and ankylosing spondylitis (AS) patients on IFX originator therapy. *BASDAI* bath ankylosing spondylitis disease activity index, *DAS28-ESR* disease activity score 28-joint count, *ESR* erythrocyte sedimentation rate, *NAB* neutralizing antibody

Demographics	CD	UC	RA	PsoA	AS
Number of total included patients (%)	73/125 (58.4)	28/125 (22.4)	9/125 (7.2)	5/125 (4)	10/125 (8.0)
Age (years)	46.2 ± 15.3	46.0 ± 18.0	59 ± 10.1	59.2 ± 14.2	52.2 ± 9.5
Female participants (%)	58.9	46.4	66.7	80.0	30.0
Baseline characteristics					
Infliximab treatment duration (years)	3.9 ± 1.4	3.5 ± 1.4	3.6 ± 1.4	2.9 ± 1.2	4.6 ± 0.5
Treatment interval of infliximab therapy (days)	46.4 ± 8.9	46.3 ± 9.1	46.2 ± 8.9	45.1 ± 9.0	45.5 ± 9.6
Concomitant immunosuppressive therapy (%)^§^	32.8	32.1	88.9	100	0
Parameters at baseline					
Patient’s assessment of disease activity Numerical scale of 1–10: 1 representing very bad, 10 representing excellent	7 (1)	8 (1)			
DAS28-ESR			3.1 (2.6)	4.0 (2.5)	
BASDAI					4.5 (2.0)
C-reactive protein (μg/mL)	1.9 (4.1)	1.5 (2.0)	1.5 (5.3)	1.0 (7.2)	1.1 (2.9)
Fecal calprotectin (μg/g)	85.0 (204.8)	108 (1077.5)			
ESR (mm/h)			14.0 (13.0)	9.0 (27.0)	15.0 (14.0)
IFX trough (μg/mL)	4.4 (4.3)	3.9 (4.1)	5.1 (9.0)	7.6 (20.5)	6.5 (8.0)
Positive IFX NABs (%)	4/73 (5.5)	1/28 (3.6)	0	0	0

^§^Azathioprine, mercaptopurine, thioguanin, methotrexate, leflunomide, and sulfasalazine. Mean ± standard deviations are presented above for normally distributed variables. *N* (%) for categorical variables. Median and (interquartile range) for non-normally distributed variables

A nocebo-effect response was defined as an unexplained, unfavorable therapeutic effect subsequent to a non-medical switch from originator infliximab to biosimilar infliximab with regaining of the beneficial effects after reinitiating the originator [8]. Nocebo effects were evaluated by the patient’s treating physician. Patients were included at different time points after the beginning of study. Nocebo response was calculated in all patients who were included in the period between July 2016 and April 2017, with an observation period of at least 6 months after the non-medical switch. The nocebo-effect rate was calculated by dividing the number of patients with a nocebo-effect response, by the number of all included patients in the described period of 9 months.

### Statistical considerations

All data are expressed as *N* (%) for categorical variables (unless specified otherwise) and as mean ± SD or median (interquartile range, i.e., difference between 75th and 25th percentiles) for numerical variables. Numerical endpoints on effectiveness and laboratory outcomes were analyzed using linear mixed models to assess their longitudinal effect, i.e., change over time upon infliximab transition. For each diagnosis, CD, UC, and ankylosing spondylitis separately and combined for psoriatic arthritis and rheumatoid arthritis, we checked which polynomial trend, i.e., cubic, quadratic, or linear trend, fitted the data best using likelihood ratio tests. If the cubic and/or quadratic trend was not significant, then a linear trend was reported. A patient-specific random intercept was included in the model to correct for repeated measures within a patient. All available data have been used to determine and maximize the likelihood [15]. The data analysis comprised collected data from baseline and after each infliximab biosimilar infusion, irrespective of whether patients become nocebo-response patient or non-responder. All analyses were performed using IBM SPSS Statistics for Windows (Version 23.0. Armonk, NY: IBM Corp.). A *P* value smaller than or equal to 0.05 was considered statistically significant.

## Results

In total, 146 patients were invited to participate in the period between July 2016 and April 2017. A group of 125 (85.6%) patients agreed and subsequently provided an informed consent and enrolled in the study. The majority, 101 patients, had IBD of whom 73 were diagnosed with CD and 28 patients with UC. The rheumatologists enrolled nine, five, and ten patients for rheumatoid arthritis, psoriatic arthritis, and ankylosing spondylitis, respectively. Demographics and baseline characteristics are depicted in Table 1. Unusual (serious) drug reactions, that is, other than known in case of originator infliximab therapy, were not reported. One patient with UC (also treated with thioguanin and a medical history comprising gallstone disease and excessive alcohol intake) without prior history of pancreatitis died because of necrotizing pancreatitis 1 month after the last infusion with infliximab biosimilar. We classified this as unrelated to infliximab use, based on clinical presentation and the documented safety profile of infliximab, and secondarily on the time lag between last infusion and this adverse event. There was no causal relationship between adverse drug reactions and/or neutralizing antibodies to infliximab formation and the batch numbers used in the preparation of the infusions.

### Responders

Nine months after the start of the project, 61 (86.3%) CD patients and 21 (78.6%) UC patients were on infliximab biosimilar therapy after a median number of four infusions for both indications.

Eight rheumatoid arthritis, five patients with psoriatic arthritis, and eight ankylosing spondylitis patients were effectively being treated with infliximab biosimilar after a median number of three, four, and four infusions, respectively. No neutralizing antibody against infliximab was observed in these rheumatological indications.

### Non-responders

Seven IBD patients ceased infliximab biosimilar therapy due to ineffectiveness based on clinical presentation and laboratory findings. This was observed in four patients with CD and three with UC after a median number of one and three infusions, respectively. All patients were switched to other than infliximab therapy. Five of these seven patients, four with CD and one with UC, ceased their therapy in the presence of neutralizing antibody formation against infliximab originator, and, subsequently against biosimilar infliximab. At the time of transition, neutralizing antibody formation against infliximab was asymptomatic (and unknown due to delay in laboratory test results). Non-responding patients were not observed in any of the rheumatological indications.

### Nocebo

Sixteen patients were designated as nocebo patients, thirteen and three patients for IBD and rheumatological indications, respectively. This resulted in an overall nocebo rate of 12.8% (16/125) and when specified for rheumatological and IBD indications, a similar rate was observed (12.5% (3/24) and 12.9% (13/101), respectively). Eight and five nocebo-response patients with CD and UC discontinued infliximab biosimilar therapy after a median number of three and four infusions. Additionally, a feeling of less exerted effect, chills during infusions, and numbness of facial skin with tingling limbs were reported in these patients. In rheumatological indications, one rheumatoid arthritis and two ankylosing spondylitis patients discontinued infliximab biosimilar therapy due to a nocebo response following a single infusion with infliximab biosimilar. A perceived diminished effect and new-onset headache were reported in these patients. All IBD and rheumatological nocebo-response patients were successfully treated with at least two additional infusions of (re-initiated) infliximab originator.

### Longitudinal trend of treatment outcome parameters

In IBD as well as rheumatological patients, no statistically significant longitudinal change in disease activity assessments, kinetics, and laboratory outcomes was found. This is elaborated in Table 2. Only patient’s assessment of disease activity in UC patients appeared to decrease in a statistically significant way (*p* = 0.027), whilst laboratory and kinetic data showed no statistically significant change over time.

**Table 2:** Overview of differences (95% CI) at a linear trend in efficacy and laboratory data per indication after switching to biosimilar infliximab compared to baseline data. Introducing a patient-specific random intercept made the correction of repeated measurements within a patient

	CD	UC	RA + PsoA^§^	AS
Patient’s assessment of disease activity numerical scale of 1–10: 1 representing very bad, 10 representing excellent	− 1.08 (−2.19–0.04)	− 2.10 (− 3.95 to − 0.25)^*^	–	–
DAS28-ESR			− 1.28 (− 3.40–0.84)	–
BASDAI			–	− 0.52 (− 4.55–3.51)
C-reactive protein (μg/mL)	− 2.20 (− 8.09–3.69)	4.28 (− 0.84–9.40)	− 3.39 (− 9.92–3.14)	0.12 (− 8.52–8.76)
ESR (mm/h)	–	–	0.92 (− 14.53–16.38)	− 5.24 (− 12.60–2.12)
Fecal calprotectin (μg/g)	− 256.62 (− 760.72–247.47)	− 370.53 (− 1814.85–1073.80)		
IFX trough (μg/mL)	0.61 (− 3.77–4.99)	1.12 (− 0.50–2.74)	4.11 (− 5.72–13.94)	− 1.18 (− 7.01–4.64)

^*^A *p*-value smaller than 0.05 was found and considered as statistically significant. These data comprises measurements in responder, non-responder and nocebo-effect response patients. Differences found are expressed as mean annual change. Crohn’s disease (CD), ulcerative colitis (UC), rheumatoid arthritis (RA), psoriatic arthritis (PsoA), and ankylosing spondylitis (AS).

^§^Data in these indications are reported as one due to limited patient sample sizes per indication

## Conclusion and discussion

In this pragmatic trial, we monitored effectiveness and safety in immune-mediated inflammatory disease patients, who switched from originator infliximab to biosimilar focusing on nocebo response, together with a biosimilar implementation strategy to optimize a shared therapeutic decision with individual patients. Core element of decision-making between doctor and patient was an informed consent, allegedly to decrease nocebo response next to empowerment of patients. Infliximab batch-number information was registered to objectify individual therapeutic findings for idiosyncratic or medication batch-specific effects, beyond the general statistical data.

Similarity in effectiveness, antibody-to-infliximab, and the adverse drug reaction profile was corroborated in these series consistent with findings from others [2, 3, 16, 17]. We observed an overall nocebo-response rate of 12.8% that not differed between IBD and rheumatological patient groups. As a consequence of a single center study, small numbers of patients with rheumatological indications were included in this series. Although commonly not quantified or reported by means of objective disease outcome measures, non-medical switching may still have a negative impact on patient’s perceived disease burden. The NORSWITCH study, a 52-week randomized clinical non-inferiority trial that compared originator infliximab to biosimilar included IMID patients and reported disease flare rates after switch of 26 and 30% for originator and biosimilar, respectively [3]. These numbers may reflect nocebo effect as a result of non-medical switching, at least in part, however, the authors did not specify this. The percentage of patients with a nocebo response observed in this study with a mixed IMID population is higher than that reported in the observational non-medical infliximab switch study of the DANBIO registry considering rheumatological patients [16]. The authors only reported a 3.4% lower rate in drug retention for patients on biosimilar infliximab after 413 days when compared to originator infliximab. This observation was attributed to a possible nocebo effect. Our findings are in line with other real-world data provided by an observational single clinic study performed in Norway [18]. In this study, 39 rheumatoid arthritis and ankylosing spondylitis patients on originator infliximab were switched to biosimilar and a nocebo-response effect of 15% after a median of 11 months was reported [18].

Although our study is not controlled for measuring the nocebo-response effect of shared decision-making, we hypothetically propose that patient empowerment may decrease nocebo-response rate, whilst effectiveness and safety are maintained. Furthermore, extra safety provided by intensive monitoring on individual basis may give non-medical biosimilar switching an additional gain.

The objective of implementing biosimilars is economically driven [19]. In our series, for example, the estimated loss of financial gain by transition into biosimilar is not only influenced by the described nocebo-effect rate. The percentage of patients not willing to switch (14.4%), the nocebo-effect rate (13%) together with implementation costs drove the overall drug cost reduction on a population level below 37% given a biosimilar price reduction of 50% by no originator drug discount.

We therefore suggest that full implementation of biosimilars in naïve patients could be an attractive economical strategy without further risking non-specific treatment effects in certain patient groups by switching [19]. This particularly holds true for biologicals with a limited period of use. Infliximab, indeed seems to be used for long periods in only few patients, treatment discontinuation rates are approximately 15% per year in the first years after starting infliximab therapy in IBD as well as rheumatology indications [16, 20].

The objective of the described biosimilar implementation was to obtain individualized treatment decisions based on actuary data on effectiveness and safety provided by a registry. We observed nocebo effects and propose its use as a treatment quality assessment of patient’s acceptance in any switch concerning biosimilars or second-line biologics. Prospective evidence is warranted to conclude if shared decision-making and patient empowerment could reduce the nocebo-effect rate. Further research comprising larger data sets in particular with patient-reported outcomes is needed to elucidate which patient aspects influence the nocebo effect in order to stratify for patients who are more prone to the effect. Although the main objective was not to study infliximab, biosimilar effectiveness and safety the amount of included patients, especially in rheumatological indications, can be regarded as a limit.

In conclusion, until now, there is sparse data on the practical implementation of non-medical switching and for biosimilar transition in particular. In our series with IBD and rheumatological patients, we demonstrated effectiveness and safety on the transition into biosimilar infliximab, whilst using a protocol involving informed consent from patients for non-medical switching. Success of non-medical biosimilar switch in terms of beneficial clinical, pharmacological, and even economical effects are relevantly/critically influenced by the nocebo-response effect rate.

In addition, a quality database registration on effectiveness, safety, kinetics, laboratory parameters, and medication-batch data seemed pivotal for follow-up of changes in medication strategy and safety on both individual as well as population level regarding biosimilar transition, reflecting an accurate nocebo assessment and quantification. This also contributed to shared decision-making and individualized medicine.

## Electronic supplementary material

ESM 1 (DOC 43.0 kb)
