# Supplementary material for: The nocebo effect challenges the non-medical infliximab switch in practice
Source: Eur J Clin Pharmacol. 2018 Jan 24;74(5):655–61. doi: 10.1007/s00228-018-2418-4 (PMC5893662; doi:10.1007/s00228-018-2418-4)
Supplement: Supplementary file 1 — (DOC 43.0 kb) [file 228_2018_2418_MOESM1_ESM.doc]

Authors’ translation from Dutch into English

| Date: June 2016 |
| --- |
| ID: ld/1606X |
| Subject: Change of infliximab brand; Remicade® becomes Inflectra® |

Dear Sir, dear Madam,

You are currently receiving the drug infliximab (brand name Remicade®) on the day-care infusion centre of the Zuyderland Medical Centre. In February 2015, another infliximab with the brand name Inflectra® was launched on the market. This product is as good, safe and effective as the infliximab with the brand name Remicade® you are receiving now, a so-called similar.

In this letter you will find out why the Zuyderland Medical Centre is currently intending to switch to another brand of Infliximab, what that means to you, and how we will guide you if you have any questions after reading this letter.

**Why is Zuyderland Medical Centre switching to another brand of infliximab?**

There has recently been much dialog about the possibility of using copy-drugs infliximab (biosimilars) to treat IBD. Since the introduction of infliximab in 2000, Dutch hospitals have been responsible for purchasing this effective and expensive medicine. The patent on Remicade® expired at the beginning of 2015.

The Zuyderland Medical Centre, together with the medical specialists, opts for the most effective and safe medicines. If there are medicines of comparable quality and safety, we also take the price into account. In this case, the quality of Remicade® and Inflectra® is comparable. Both preparations contain the same active ingredient; this is infliximab. Comparability has been demonstrated after extensive testing and therefore Inflectra® has also been approved by the medicines agencies EMA (in a European context) and CBG (in the Netherlands). The big price difference between Inflectra® and Remicade® makes us choose for Inflectra®. By opting for Inflectra®, we help to ensure that care remains affordable in the future.

**What does this mean for you?**

In September 2013, Inflectra® (from Pfizer) was approved and registered for the same indications as Remicade® (produced by MSD) in Europe and it is now successfully and widely used in other Dutch and foreign hospitals. In Zuyderland MC, we already have a positive clinical experience with the introduction of Inflectra® including patients with inflammatory bowel disease or rheumatic disease. The quality, effectiveness and safety are well researched and comparable to Remicade®. There is no medical reason to believe that a change from Remicade® to Inflectra® will have consequences for your treatment.

Nevertheless, we think it is important that you are being supported in the transition. Therefore, prior to the 1st infusion and during subsequent administrations, we check the inflammation values in your blood and the concentration of the drug and any antibodies against the drug. A tube of blood is collected from you via the infusion connection by the nurse during the infusion visit at the daycare ward. You will not be additionally venapunctured for this purpose.

*For IBD patients only;*

At four moments during the upcoming infusions you will also be asked to bring along a relief sample, following instructions.

In addition, you will be asked to complete a questionnaire so that we can monitor your disease activity upon transition. Your gastroenterologist / rheumatologist supports the policy of infliximab-Remicade into infliximab-Inflectra transition in the best possible conditions.

**Practical matters of interest**

We value your informed consent. If you are in agreement with the conversion of Remicade® into Inflectra® by means of a guided transition, you can let us know by sending an e-mail message to: [ibd@zuyderland.nl](mailto:ibd@zuyderland.nl) / [reumatologie@zuyderland.nl](mailto:reumatologie@zuyderland.nl)

If you would like to be further informed by telephone, you can also let us know by sending an e-mail message to: [ibd@zuyderland.nl](mailto:ibd@zuyderland.nl) / [reumatologie@zuyderland.nl](mailto:reumatologie@zuyderland.nl)

If you already have an outpatient appointment, your gastroenterologist / rheumatologist will discuss the transition into Inflectra® with you during the next outpatient visit.

If you do not have a new outpatient appointment scheduled and you do not want a new appointment, please send an e-mail to [ibd@zuyderland.nl](mailto:ibd@zuyderland.nl) / [reumatologie@zuyderland.nl](mailto:reumatologie@zuyderland.nl). In response to this e-mail, the outpatient clinic will contact you to schedule a new appointment on which the transition to Inflectra® will be discussed with you.

*For IBD patients only;*

Before proceeding with Inflectra®, we will kindly ask you to take a relief sample to the infusion centre. Please use the supplied sample container.

As an appendix we will send you a question and answer document prepared by the Dutch Medicine Evaluation Board^1,2^. This document answers frequently asked questions about the conversion of Remicade® to Inflectra®.

If you have any questions after reading this letter, please contact the undersigned by telephone or email.

Yours sincerely,

dr. X, gastroenterologist / rheumatologist

email x@zuyderland.nl

tel 088-4597800

1 Vragen en antwoorden over biologische geneesmiddelen – informatie voor patienten en consumenten (“Question and Answer overview”). Availbale at; <https://www.cbg-meb.nl/documenten/brochures/2016/04/08/vena-biologische-geneesmiddelen-informatie-voor-patienten-en-consumenten> (accessed 4 January 2018).

2 What I need to know about Biosimilar Medicines – Information for patients. Availbale at; <http://ec.europa.eu/DocsRoom/documents/20961> (accessed 7 January 2018).
